# Clinical and pathological observation of conversion therapy for malignant peritoneal mesothelioma: a case report and literature review

**DOI:** 10.3389/pore.2023.1611577

**Published:** 2024-01-08

**Authors:** Minying Deng, Xinyi Zhang, Chen Xu, Rongkui Luo, Lingli Chen, Yuhong Zhou, Yingyong Hou

**Affiliations:** ^1^ Department of Pathology, Zhongshan Hospital, Fudan University, Shanghai, China; ^2^ Department of Medical Oncology, Zhongshan Hospital, Fudan University, Shanghai, China

**Keywords:** peritoneal malignant mesothelioma, conversion therapy, clinical pathology, delayed interstitial pneumonitis, PD-L1 expression

## Abstract

**Background:** Malignant mesothelioma (MM) is a tumor originating from the pleura, peritoneum, or pericardial cavity. It is divided into diffuse and localized malignant mesothelioma, with four subtypes in diffuse MM: epithelioid, sarcomatoid, desmoplastic, and biphasic, with biphasic being less common. The onset of this tumor is insidious, and the prognosis is extremely poor in some cases, with a median survival of 6–18 months and no standard treatment options in the past.

**Aims:** We report a case of peritoneal malignant mesothelioma that was successfully treated with transformative therapy. We also review the literature in the hope of providing reference for the treatment and pathological diagnosis of such patients.

**Methods:** The case of the peritoneal malignant mesothelioma was processed and reported in the routine manner for biopsy specimens at different stages.

**Results and conclusion:** We report a case of a malignant tumor originating in the hepatorenal recess, which was diagnosed as biphasic malignant mesothelioma through a biopsy. Immunohistochemical testing showed PD-L1 expression. After multidisciplinary discussion, the patient received transformative treatment, including a trial of combined immunotherapy. The tumor significantly shrank, and the patient obtained a chance for curative surgical resection. Microscopic examination showed significant collagenization in the lesion area, with almost no residual tumor. After 19 months of comprehensive treatment, the patient developed multiple fluffy opacities under the pleura of both lungs. Transthoracic core needle biopsy under CT guidance, the pathology showed organizing pneumonia, considering it as delayed interstitial pneumonitis due to immunotherapy based on previous treatment history. Successful comprehensive treatment was achieved for this case of peritoneal malignant mesothelioma, and the patient has been alive without evidence of disease for 33 months, with long-term follow-up. In this process, the pathologist had three opportunities for pathological diagnosis, which required understanding the patient’s medical history, being attentive to the clinical purpose of the specimen, and providing accurate responses to morphological changes at different stages, along with corresponding descriptions and diagnoses to provide effective information for clinical treatment.

## Introduction

Malignant mesothelioma (MM) is a highly invasive and difficult-to-cure tumor that is relatively rare, accounting for approximately 0.3% of all malignant tumors; its peritoneal manifestation is even rarer [1]. In recent years, immune checkpoint inhibitors (ICIs) in immunotherapy have significantly improved the prognosis of a variety of solid tumors, including malignant pleural mesothelioma, but there is no standard and effective treatment for malignant peritoneal mesothelioma (MPM). In this case, pathological remission was achieved after comprehensive treatment of MPM, and late-onset PD1-related immune pneumonia occurred. With the application of new treatment strategies in the clinical treatment of malignant mesothelioma, pathologists may have the opportunity to encounter similar scenarios of morphological changes after treatment and secondary changes in other organs. Due to the rarity of this situation reported in this article, we hope to provide a reference for the treatment and pathological diagnosis of such patients.

## Case reports

**FIGURE 1:**
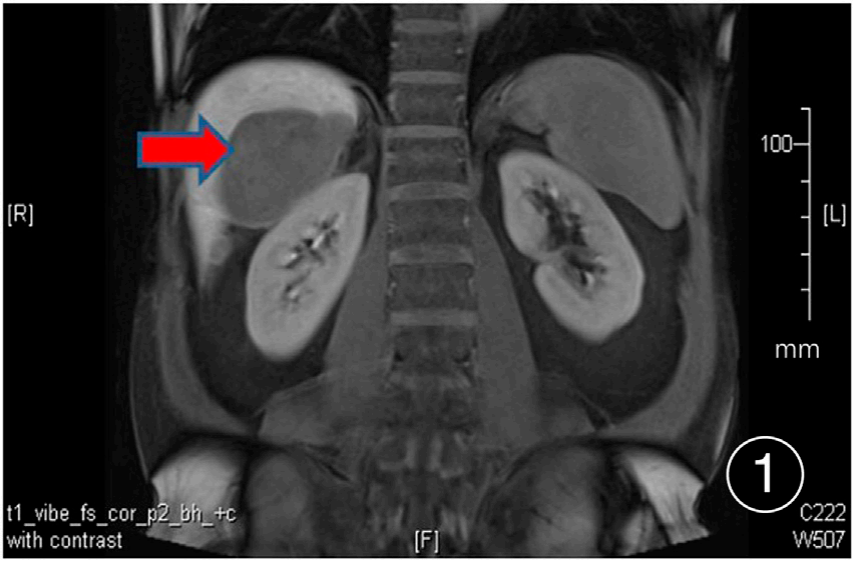
PET/CT shows multiple nodular and lump-like abnormal signal lesions under the liver surface, the larger one is located in the space between the liver and the right kidney, about 85 mm × 57 mm in size.

**FIGURE 2:**
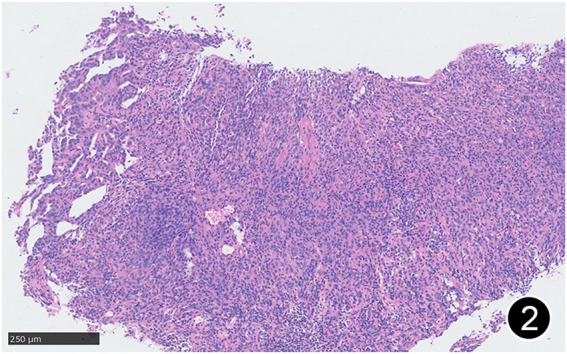
Some areas of MPM are arranged in nests and sheets, and some areas have glandular structure. This is a mid-power magnification of Hematoxylin and Eosin (HE) staining.

**FIGURE 3:**
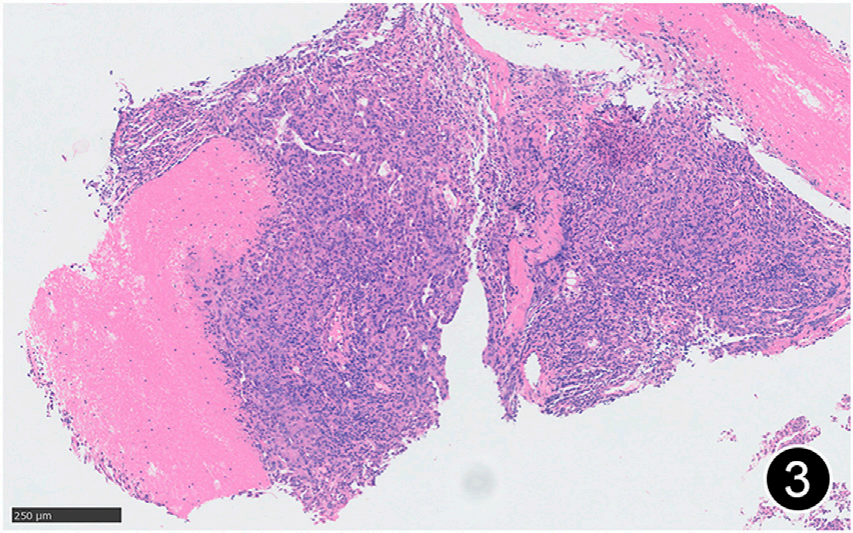
Necrosis can be seen in some areas of MPM in this mid-power magnification of HE staining.

**FIGURE 4:**
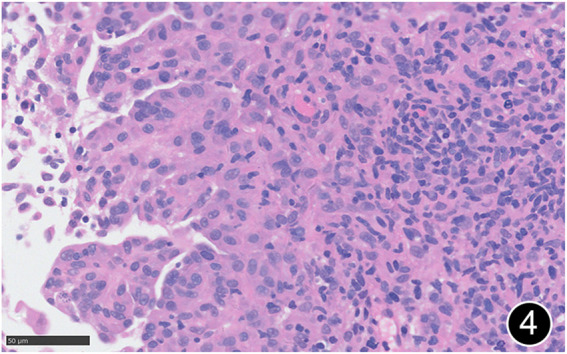
The tumor cells are epitheliod and have abundant cytoplasm. This is a high-power magnification of HE staining.

**FIGURE 5:**
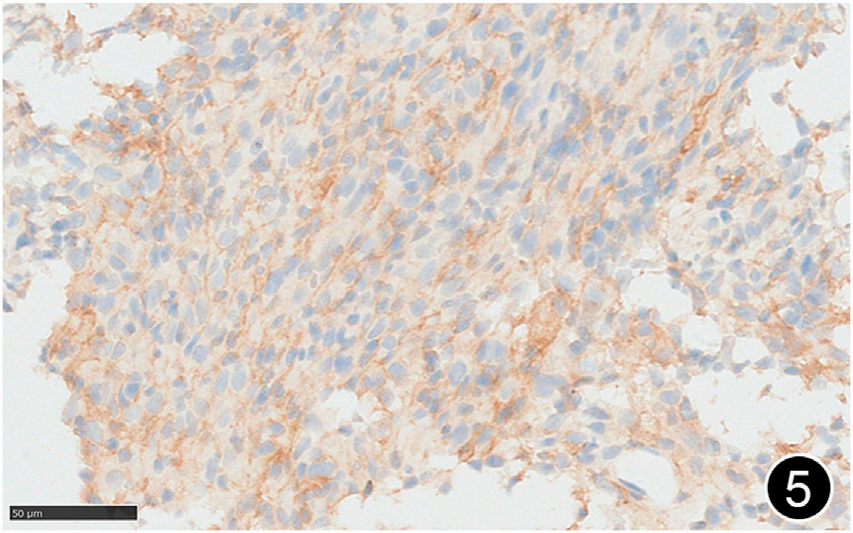
The tumor cells diffusely express PD-L1 clone 288, a high-power magnification of the EnVision method.

**FIGURE 6:**
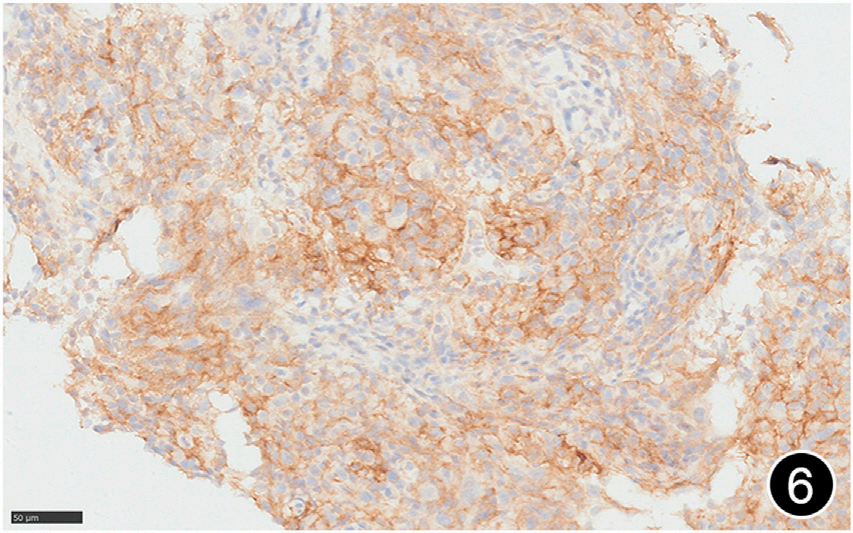
The tumor cells diffusely express PD-L1 clone E1L3N, a high-power magnification of the EnVision method.

**FIGURE 7:**
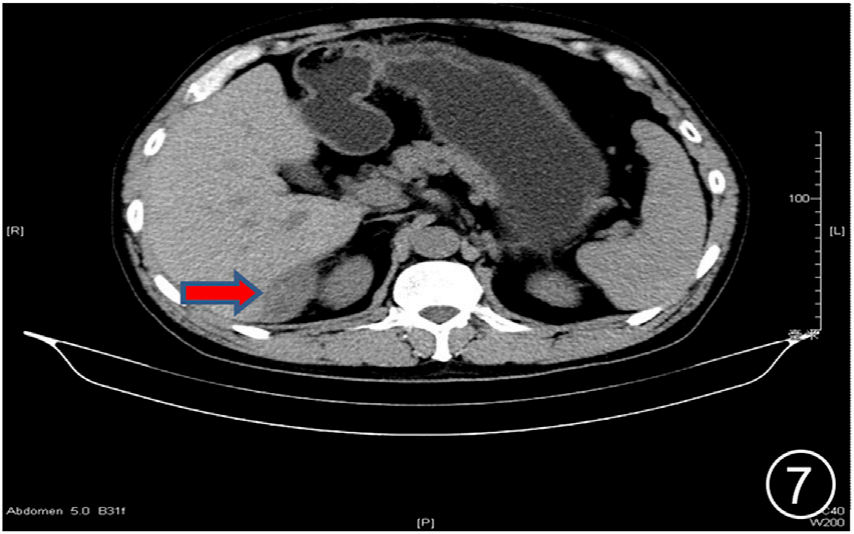
After six rounds of chemotherapy and four rounds of immunotherapy, the repeat CT showed uneven liver surface and the lesion in the liver-kidney space was significantly smaller than before.

**FIGURE 8:**
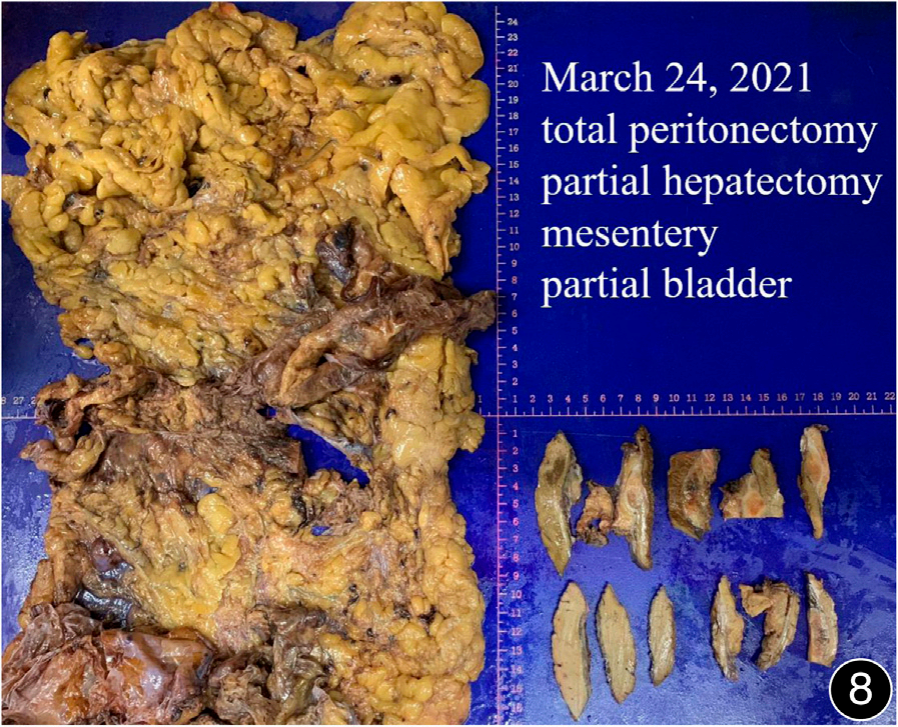
After six rounds of chemotherapy and four rounds of immunotherapy, a total peritonectomy was performed, and possible nodules were seen on the cut surface of the liver.

**FIGURE 9:**
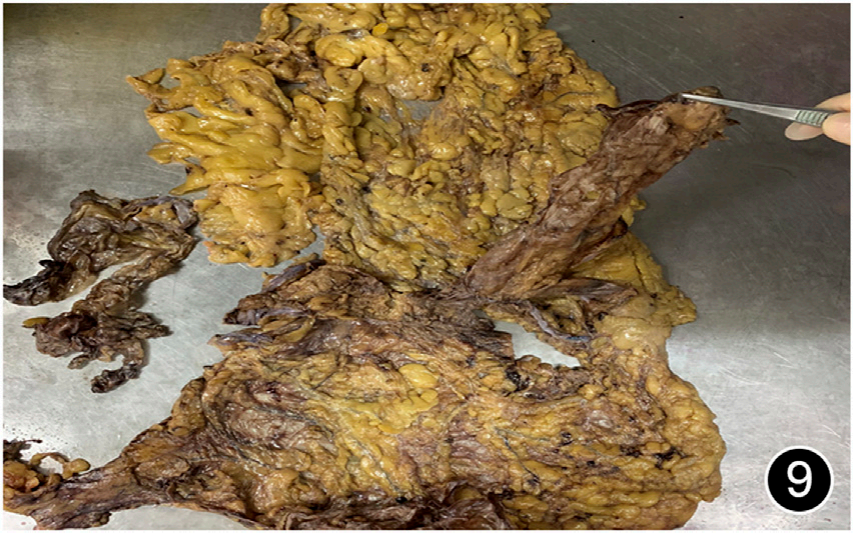
After six rounds of chemotherapy and four rounds of immunotherapy, a total peritonectomy was performed, and hard nodules were seen in the omental tissue.

**FIGURE 10:**
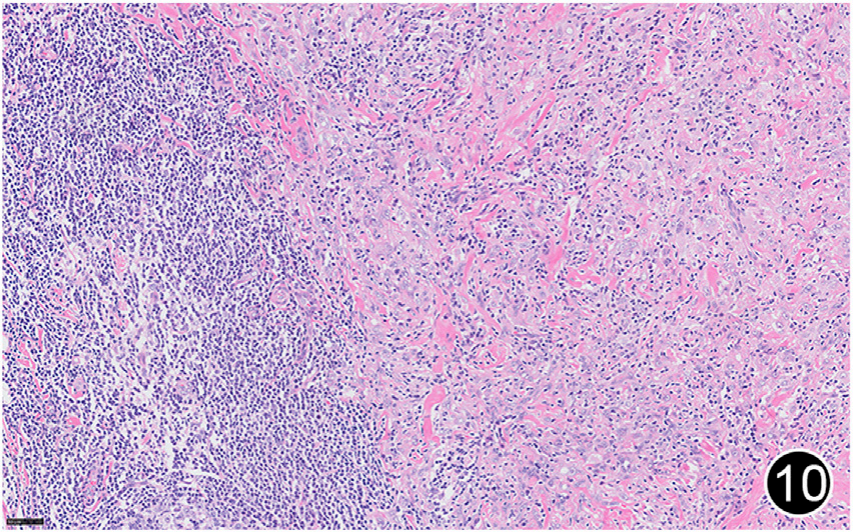
After six rounds of chemotherapy and four rounds of immunotherapy, the tumor regressed significantly, with a large amount of lymphocytic infiltration around the residual tumor cells. This is a high-power magnification of HE staining.

**FIGURE 11:**
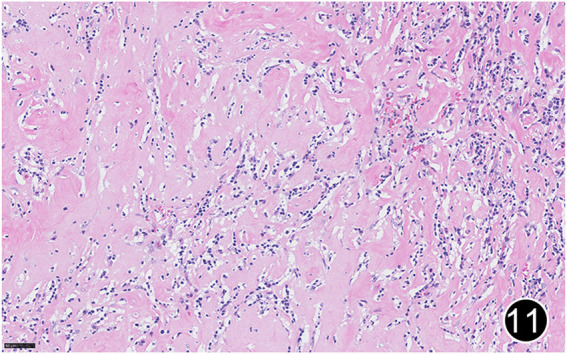
After six rounds of chemotherapy and four rounds of immunotherapy, the tumor regressed significantly, with significant collagenization in the lesion. This is a high-power magnification of HE staining.

**FIGURE 12:**
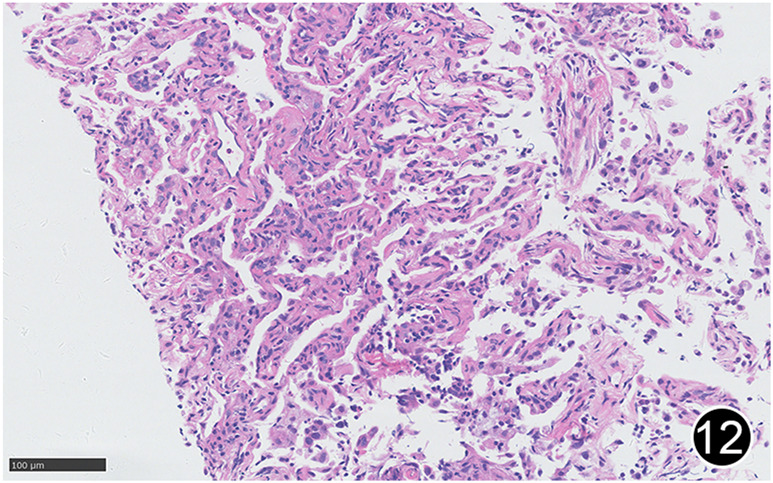
After six rounds of chemotherapy and four rounds of immunotherapy, there was a small amount of lymphocytic infiltration in the lung interstitium, the alveolar septum was slightly widened, and there was a small amount of lymphocytic infiltration in the alveolar cavity, forming changes of organizing pneumonia. This is a high-power magnification of HE staining.

The patient, a 61 years-old male, had no history of asbestos exposure. He was admitted to our hospital on 30 October 2020, due to the discovery of a liver mass during a physical examination. PET/CT showed multiple liver metastases beneath the liver capsule (the largest measuring 85 mm × 57 mm), with local compression and decreased function in the right posterior lobe of the liver. There were also multiple peritoneal implant metastases, and a possible metastasis next to the thoracic/dorsal 11th vertebra on the right side (Figure 1). On 2 November 2020, a biopsy was performed, and the pathology showed a malignant tumor of epithelioid cells with necrosis. Extensive immunohistochemical analysis confirmed the diagnosis of malignant mesothelioma. Some areas showed papillary and glandular structures, while other areas showed nest-like structures, accompanied by significant collagenization in the stroma, classified as biphasic type (Figures 2–4). Immunohistochemical staining results showed the tumor cells expressing CAM5.2, broad-spectrum CK (CKpan), Vimentin, Desmin, CK7, CK8, WT-1, D2-40, Calretinin, P63, and EMA, while not expressing a-SMA, S-100, ARG-1, SOX10, CD117, DOG-1, CD21, CD23, CD35, HBME-1, A103, P40, Hepa. P16 loss of expression was observed, and the Ki67 proliferation index was approximately 60%. PD-1 was positive in the stroma with abundant lymphocytes, while PD-L1 clone 28-8 was positive in tumor cells (70%) and negative in stromal cells. PD-L1 clone E1L3N was positive in tumor cells (70%) and negative in stromal cells. PDL1 clone 22C3 was positive in tumor cells (5%) and negative in stromal cells (Figures 5, 6). Dual-color fluorescence *in situ* hybridization (FISH) showed that approximately 10% of tumor cells exhibited monosomy of chromosome 9, approximately 5% had heterozygous loss of the P16 gene, and approximately 2% had homozygous loss of the P16 gene. After the pathological diagnosis was confirmed, considering the involvement of multiple sites including the peritoneum, liver, hepatorenal recess, bladder, and diaphragm, a multidisciplinary discussion was conducted, and transformative treatment was planned. The treatment plan was fully communicated with the patient, and combined immunotherapy was attempted. Starting from 10 November 2020, the patient received the first cycle of treatment, which included pembrolizumab (1,000 mg, day 1) and cisplatin (75 mg, day 1–2) chemotherapy, along with supportive treatments such as dexamethasone, palonosetron, apatinib, atorvastatin, and lansoprazole. On 3 December 2020, the patient received the first dose of pembrolizumab (200 mg, intravenous drip, every 3 weeks). Until 17 March 2021, a total of six cycles of chemotherapy (pembrolizumab + cisplatin) and four cycles of immunotherapy (pembrolizumab) were administered. CT scans showed a significant reduction in the size of the lesions compared to before (43 mm × 27 mm) (Figure 7). NSE and CA125 markers improved. Therefore, on 24 March 2021, the patient underwent a total peritonectomy, partial hepatectomy, and intraperitoneal hyperthermic chemotherapy (cisplatin and doxorubicin). During the operation, scattered thickened lesions and noticeable retraction were observed in the peritoneum (Figures 8, 9). The postoperative pathology showed that the lesions were distributed on the surface of the liver and peritoneum, with significant collagenization in the lesions, almost complete disappearance of tumor tissue, and significant infiltration of lymphocytes in some lesions. Based on the morphology and immunohistochemistry results, residual tumor cells were less than 5% and exhibited significant wrinkling and thinning (Figures 10, 11). Dual-color FISH did not show definite evidence of P16 gene double deletion, and the FISH results tended to be negative. On 10 May 2021, CEA, CA125, and NSE were within normal ranges during hospital follow-up, and abdominal CT scan did not show any definite residual tumor activity. The patient continued PD-1 immunotherapy until 9 May 2022, for a total of 22 cycles. On 30 May 2022, chest CT showed pneumonia-like lesions in the middle and lower lungs, possibly drug-induced organizing pneumonia. Therefore, on 6 June 2022, a lung biopsy was performed under CT guidance, and the pathology showed slight fibrous tissue proliferation in the lung tissue and bronchial wall, slightly widened interstitial spaces, focal protrusions into the alveolar cavities, and localized organizing pneumonia changes, consistent with organizing pneumonia. Based on the imaging and pathological findings, PD-1-related immune pneumonia was considered, with the lesion area being less than 30% of the lung tissue area, classified as grade I (Figure 12). Immunotherapy was discontinued, and targeted treatment with olaparib was continued. CT scans showed improvement in the pneumonia in both lungs compared to before. The patient was followed up regularly until 26 July 2023, and no recurrence or metastasis was observed.

## Discussion

MM was first described by Miller and Wynn in 1908 [2]. Data show that there are about 3,300 new cases of MM in the United States each year [3]. The most common sites of occurrence are the pleura, followed by the peritoneum, accounting for about 10%–15%. MPM is even rarer, with a global incidence rate of approximately 0.2/1,000,000–3/1,000,000 [4]. MPM is caused by multiple factors, such as genetic factors, past radiation history, talcum powder, aspergillus, exposure to radiation, mica, Hodgkin’s disease and SV40, but the most important of these is asbestos exposure, accounting for about 90% [5–10]. According to related research reports, it is estimated that between 2005 and 2050, there will be a cumulative total of about 94,000 cases of malignant pleural mesothelioma and 15,000 cases of MPM in the United States [11]. Currently, the incidence of MM is highest in the United Kingdom, Australia and New Zealand, while it is lowest in Japan and Central European countries. However, Russia, China, India and Brazil, all major manufacturing countries, used asbestos considerably in the last century. This fact, which has not been given enough attention, could pose a significant public health problem in the future.

Clinically, MPM is more common in males and the age of onset ranges from 4 to 31 years, especially in patients with occupational exposure. Its clinical manifestations are non-specific, mainly manifesting as abdominal distension (31%–87%), abdominal pain (7%–31%), abdominal mass (17%), fever (15%), vomiting (31%), diarrhea (12%), etc. [9, 12–14]. Timely recognition of the disease is difficult due to a lack of effective methods for early diagnosis; MM is generally confirmed in the late stage, and has a long latent period of 40–50 years, which increases the diagnostic difficulty and hinders early treatment. This case was detected during a physical examination, without any symptoms, and has already metastasized to the liver when detected, consistent with literature reports.

Grossly, MPM can present as multiple small nodules or plaques on the peritoneal surface, mesentery, or omentum, which can fuse and extend into the fissures, but it is mostly limited to the internal cavity and rarely involves intra-abdominal and extra-abdominal organs. Histologically, the 2015 WHO classification of thoracic tumors divides MM into diffuse and localized MM, which are further divided into epithelioid, sarcomatoid (fibrous), and biphasic (mixed) types, of which the epithelioid type is most common, accounting for 60%–80%. The subtype with the best prognosis is the epithelioid, which includes tubular papillary, solid, trabecular, micropapillary, glandular cystic, clear cell, deciduoid, small cell, signet ring cell, and mucinous subtypes. The sarcomatoid type accounts for about 10%, and its subtypes include conventional, desmoplastic, with heterologous osteosarcomatous elements, and lymphohistiocytoid variants, which have the worst prognosis. The biphasic type (10%–15%) consists of both epithelioid and sarcomatoid subtypes, requiring each subtype to account for at least 10%. This case conforms to biphasic MPM.

Morphologically, MPM varies widely and needs to be differentiated from peritoneal metastasis of primary tumors of the gastrointestinal tract, malignant melanoma, lymphoma, ovarian cancer, serous peritoneal carcinoma, and epithelioid hemangioendothelioma. However, the results of immunohistochemical staining provide some hints, but there is no single index of high specificity and sensitivity at present. Therefore, pathologically, a combination of positive mesothelioma markers, such as calretinin, CK5/6, WT-1, D2-40, and negative mesothelioma markers, such as CEA, MOC31, Ber-EP4, PAX8, CD15, TTF1, B72.3 is often used. The guidelines suggest at least two positive markers and two negative markers to assist in the diagnosis and differential diagnosis of MPM [15].

MPM has unique molecular genetic characteristics. Literature reports that the most common gene mutation in pleural mesothelioma is the inactivation of the CDKN2A/B locus on 9p21, as high as 80%, however, it is relatively rare in MPM, about 8% [16]. Some scholars have performed large panel sequencing on 13 MPM patients, with results showing that 9 cases had BAP1 bi-allelic inactivation, 2 cases had BAP1 single-allele loss. Additionally, 2 cases of NF3 mutation, 3 cases of SETD2 mutation, and 2 cases of DDX3X mutation were detected [17]. Offin et al. [16] reported consistent research results, with Tp53 and LATS2 gene mutations also observed, and suggested that patients with BAP1 mutations or expression loss had shorter survival times. In 2013, Panagopoulos et al. [18] first reported the EWSR1-YY1 fusion gene in MPM, but its specific prevalence and mechanism of action in MPM still need further exploration and research.

MPM has a poor prognosis, and early diagnosis is difficult. The median overall survival time is 8 months, the 5 years overall survival rate is 17%–33%, and the 10 years overall survival rate is 9% [19, 20]. Currently, there is no unified standard treatment plan. In recent years, the treatment of MPM has developed from a single treatment method to a combination of multiple treatment methods. Currently, for operable patients, cytoreductive surgery (CRS) combined with hyperthermic intraperitoneal peroperative chemotherapy (HIPEC) is considered the standard treatment for MPM. Feldman et al. [21] conducted a phase II clinical trial, with 49 MPM patients, and found that CRS combined with HIPEC treatment can extend the median overall survival time of patients to 92 months, with a 5 years overall survival rate of 59%. However, the high perioperative mortality rate (0%–6%) and morbidity rate (15%–56%) need to be emphasized [22]. Systemic chemotherapy is the first choice of treatment for inoperable tumors or recurrent tumors or patients who do not wish to have surgery. Some researchers believe that pemetrexed combined with cisplatin chemotherapy, compared with pemetrexed monotherapy, has a longer median overall survival time (13.1 months VS 8.7 months), with a total remission rate of about 25% [23, 24]. However, attention should also be paid to the occurrence of grade III/IV adverse events after chemotherapy, such as dehydration, nausea and vomiting. In recent years, emerging therapies such as immunotherapy and anti-angiogenesis targeted therapy have also entered the clinical trial stage. In December 2020, the U.S. Food and Drug Administration (FDA) announced the use of the immunocombo therapy of nivolumab monotherapy and ipilimumab monotherapy for the treatment of untreated unresectable non-epithelioid malignant pleural mesothelioma in adult patients, and it has been approved in China. However, there is no data to support the effective treatment of MPM. Therefore, Raghav et al. [25] evaluated the efficacy and safety of the combination of PD-L1 (atezolizumab) and VEGF (bevacizumab) blockers (AtezoBev) in 20 patients with advanced and unresectable MPM, especially those with progression or intolerance to platinum-based pemetrexed chemotherapy. The median duration of remission was 12.8 months, the 1 years progression-free survival rate and overall survival rate were 61% and 85%, respectively, and it was believed that the effect of atezolizumab and bevacizumab on the remission rate and survival rate in advanced peritoneal mesothelioma patients who had previously received chemotherapy exceeded the expected results of conventional therapy. Hassan et al. [26] conducted a prospective study to evaluate the efficacy of avelumab in 53 patients with unresectable pleural or peritoneal mesothelioma who had previously received platinum-based and pemetrexed chemotherapy and experienced disease progression. The study showed that avelumab had durable anti-tumor activity, effectively controlled disease progression, and had acceptable side effects and safety. However, the authors did not mention the specific response of pleural mesothelioma or peritoneal mesothelioma. Fennell et al. [27] also conducted a prospective study on 332 patients with pleural or peritoneal mesothelioma who had previously received first-line platinum-based chemotherapy and experienced disease progression. Among them, 39 patients had peritoneal mesothelioma, with 26 receiving nivolumab treatment and 13 receiving placebo treatment. The results showed that the patients with recurrent peritoneal mesothelioma who received anti-PD-1 therapy had better progression-free survival and overall survival compared to the placebo treatment group. Increasing evidence suggests a higher expression of PD-L1 in MPM [28]. The MESOPEC trial is also underway to assess the feasibility and safety of dendritic cell-based immunotherapy as an adjuvant treatment for MPM patients after CRS-HIPEC, and to determine if the aforementioned immunotherapy can induce a specific immune response against the tumor [29]. Some researchers have found that epithelioid MPM expresses PD-L1, and the expression of PD-L1 (clone E1L3N) in immune cells is an important independent prognostic factor for OS and DFS in patients with epithelioid MPM [30]. However, attention should also be paid to the occurrence of a series of immune-related adverse events associated with the treatment of tumors with immune checkpoint inhibitors, such as cardiac toxicity [31]. In this case, the tumor cells highly expressed PD-L1 clone 28-8 and PD-L1 clone E1L3N, and the comprehensive treatment method of neoadjuvant chemotherapy, immunotherapy, surgery and HIPEC was adopted. The tumor significantly regressed, but postoperative PD1-related immune pneumonia occurred. It should be noted that the patient has been followed up for 30 months without recurrence/metastasis.

From a pathological point of view, this case occurred in October 2020, and in December of the same year, the FDA approved immunotherapy for pleural malignant mesothelioma. There is no recommendation for MPM, but the expansion of indications or clinical trials initiated by researchers are in full swing. With the global storm of immune checkpoint inhibitors, there has been breakthrough progress in the treatment of many solid tumors, such as malignant melanoma, non-small cell lung cancer, and gastric cancer, but it is less common in tumors such as malignant mesothelioma, and it is uncommon to see remissions; therefore, this case is of relevant importance. In addition, adverse reactions caused by PD1 immunotherapy should be taken seriously. Other solid tumors have certain standards for pathological remission assessment after immunotherapy, but there is no experience in the pathological remission assessment of malignant mesothelioma. In the surgical resection of gross specimens, no obvious nodular objects have been found by the naked eye. Under the microscope, significant collagenization is observed, and only a few slender cell components can be seen. It is difficult to evaluate whether there is tumor residue, which can suggest that the pathological remission status after treatment is close to complete response. With the continuous development and progress of clinical treatment methods and techniques, pathologists should not just be limited to the current pathological sections, they should trace the source and integrate all aspects of the medical history to make the most accurate pathological diagnosis.

In conclusion, this case adopted a comprehensive treatment method of neoadjuvant chemotherapy combined with immunotherapy, surgery and HIPEC, which achieved good treatment results for MPM. However, during the treatment process, attention should be paid to PD1-related immune pneumonia. Early prevention and control, active monitoring, early diagnosis, and timely adjustment and treatment are crucial. This case report will provide reference for clinical doctors and pathologists in the diagnosis and treatment of this disease.

## Data Availability

The original contributions presented in the study are included in the article/supplementary material, further inquiries can be directed to the corresponding authors.
